# PEG-L-CHOP treatment is safe and effective in adult extranodal NK/T-cell lymphoma with a low rate of clinical hypersensitivity

**DOI:** 10.1186/s12885-018-4782-y

**Published:** 2018-09-21

**Authors:** Wen Zheng, Yuhuan Gao, Xiaoyan Ke, Weijing Zhang, Liping Su, Hanyun Ren, Ningjing Lin, Yan Xie, Meifeng Tu, Weiping Liu, Lingyan Ping, Zhitao Ying, Chen Zhang, Lijuan Deng, Xiaopei Wang, Yuqin Song, Jun Zhu

**Affiliations:** 1 Key laboratory of Carcinogenesis and Translational Research (Ministry of Education), Department of Lymphoma, Peking University Cancer Hospital & Institute, No.52 Fucheng Road, Haidian district, Beijing, 100142 People’s Republic of China; 2 The Fourth Hospital of Hebei Medical University, Shijiazhuang, Hebei Province China; 3 Peking University Third Hospital, Beijing, China; 4 307 Hospital of PLA, Beijing, China; 5 Shanxi Tumor Hospital, Taiyuan, Shanxi Province China; 6 Peking University First Hospital, Beijing, China

**Keywords:** Extranodal natural killer (NK)/T-cell lymphoma (NKTCL), Polyethylene glycol-conjugated asparaginase (pegaspargase, PEG-ASP), Therapy, Clinical trial

## Abstract

**Background:**

**The c**ombination of chemotherapy and L-asparaginase (L-ASP) treatment significantly increased survival rate in an adult patient with extranodal natural killer (NK)/T-cell lymphoma (NKTCL). However, hypersensitivity reactions of L-ASP in some patients limited its application. Polyethylene glycol-conjugated asparaginase (PEG-ASP) has a lower immunogenicity and longer circulating half-life than unconjugated L-ASP, and has been reported to be effective and well-tolerated in children with acute lymphoblastic leukemia. Cyclophosphamide, hydroxydaunorubicin (doxorubicin), oncovin (vincristine), and prednisolone (CHOP) is the most common chemotherapy for non-Hodgkin lymphoma. In this report, we sought to study the efficacy and safety of PEG-L- CHOP in NKTCL in adult Chinese patients.

**Methods:**

Our study is a prospective, multi-center, open-label clinical trial. Patients with newly diagnosed adult NKTCL and an ECOG performance status of 0 to 2 were eligible for enrollment. Treatment included six cycles of PEG-L-CHOP regimen. Radiotherapy was scheduled after 2–4 cycles of PEG-L-CHOP regimen, depending on the stage and primary anatomic site.

**Results:**

We enrolled a total of 33 eligible patients. All 33 patients completed 170 cycles of chemotherapy combined with radical radiotherapy. The overall response rate was 96.9% (32/33) with 75.8% (25/33) achieving complete responses and 21.2% (7/33) achieving partial responses. The overall survival (OS) at 1, 2, 3-year were 100, 90.61 and 80.54%, respectively. The major adverse effects were bone marrow suppression, reduction of fibrinogen level, liver dysfunction, and digestive tract toxicities. No allergic reaction and no treatment-related mortality or severe complications were recorded.

**Conclusions:**

PEG-L-CHOP chemotherapy in combination radiotherapy is safe and durably effective treatment for adult extranodal NK/T-cell lymphoma with fewer allergic reactions.

This study was approved by the Peking University Beijing Cancer Hospital Ethics Review Committee (reference number: 2011101104). The clinical trial registration number ChiCTR1800016940 was registered on July 07, 2018 at the Chinese Clinical Trial Registry (http://www.chictr.org.cn/index.aspx). The clinical trial was registered retrospectively.

## Background

Natural killer (NK)/T-cell lymphoma (NKTCL) is a highly aggressive cancer with a poor prognosis. NKTCL is relatively rare disease in the United States and Europe, and is more common in Asia, Central America and South America [1–4]. There are no standard therapeutic regimens for this disease [1–4]. Cyclophosphamide (CY) has been used successfully as a cytotoxic agent in the clinical treatment of several cancers. CY can directly activate NK cells or stimulate their production of soluble mediators in addition to its known direct cytotoxic effect on neoplastic and suppressor T-cells [1–4]. Patients with NKTCL have a limited response and high relapse to traditional chemotherapy and radiotherapy [4, 5]. CY, hydroxydaunorubicin (doxorubicin), oncovin (vincristine), and prednisolone (CHOP) is the most common chemotherapy for non-Hodgkin lymphoma. However, CHOP therapy has demonstrated insufficient effects on NKTCL [6, 7]. Previous studies found that chemotherapy regimens utilizing L-asparaginase (L-ASP) for NKTCL have obtained 55.6% complete response (CR), and 66.9% 5-year survival rates [8–11]. Based on the efficacy of L-asparaginase (L-ASP) as an anti- NKTCL regimen that prolonged patient survival [2], it was recommended as the first-line therapy for NK/T cell lymphoma by the National Comprehensive Cancer Network (NCCN) in 2010. L-ASP is an enzyme that hydrolyzes asparagine to aspartate and ammonia [12]. L-ASP specifically breaks down extracellular asparagine at sites of tumor which leads to protein synthesis inhibition and tumor cell apoptosis, without affecting normal cells [13, 14]. However, L-ASP is derived from *Escherichia coli* and may be recognized as a foreign protein by the human immune system, as evidenced by the fact that the incidence of allergic reactions to L-ASP treatment is approximately 30%. Some severe allergic reactions can be life-threatening and potentially promote the development of drug resistance [15, 16]. Additionally, L-ASP has a short half-life of about 20 h, requiring frequent daily or every other day dosing for seven consecutive days to maintain adequate serum concentrations of the drug [15]_._ To overcome the issues of allergenicity and frequent dosing, native *Escherichia coli* derived L-ASP was conjugated to polyethylene glycol to formulate polyethylene glycol conjugated asparaginase (PEG-ASP). PEG-ASP has lower immunogenicity and higher circulating half-life compared to L-ASP [15, 17–19]. PEG-ASP was approved for treatment of acute lymphoblastic leukemia (ALL) patients allergic to L-ASP by US Food and Drug Administration (FDA) in 1994, and it has been used as the first-line treatment for adult and pediatric ALL since 2006 [17]. In China, PEG-ASP was approved as the first-line treatment for pediatric ALL in 2009. There have been several reports about PEG-ASP used in combination with different chemotherapeutic regiments for the treatment of NKTCL. These studies relied on relative small patient cohorts and reported variable treatment outcomes. The overall response rate (ORR)was about 60–100% and the complete response (CR) rate was 25–83.3% in these studies [20–24]. However, the safety and efficacy of PEG-ASP-based regimens in the treatment of NK/T cell lymphoma in the Chinese population is still not well assessed. In this report, we performed a prospective, open-label, non-randomized clinical trial to evaluate the safety and efficacy of PEG-ASP in combination with standard CHOP and radiation therapy for the treatment of NK/T cell lymphoma in adult Chinese patients.

## Methods

### Study design

This study is an open-label, prospective clinical trial. The PEG-L-CHOP regimen was introduced to selected patients every 21 days for six cycles. Adverse reactions were assessed each cycle and efficacy was assessed every two cycles. Localized radiotherapy for lymphoma was performed after 2–4 cycles of chemotherapy primary based on lymphoma stage and location. The Peking University Institutional Ethics Review Board (IRB) approved this study and the protocol.

### Patients

Patients newly diagnosed with adult extranodal NKTCL based on histomorphology and immunohistochemistry defined by WHO criteria were eligible for the clinical trial. Inclusion criteria included at least one measurable lesion, age 18 ~ 70 years, male or female, The Eastern Cooperative Oncology Group (ECOG) performance 0~ 1, expected survival of more than three months. For females of childbearing age, only patients with a negative pregnancy test and who agreed to using effective contraceptive measures during treatment and up to one year post clinical trial were enrolled. The exclusion criteria were as follows: any invasive of lymphoma to central nervous system; pre-existing coagulation disorder concurrent neoplasms, advanced liver and kidney disease, uncontrolled cardiovascular disease within 6 months of the trial, positive HIV antibody, HBV DNA titer higher than 10^4^ copies /ml in HBsAg-positive patients post antiviral therapy; pregnant or lactating women; women of childbearing age unwilling to take contraceptive measures during the study period. The written consent including the nature of the disease, its treatment options, and its possible outcomes was informed to the patients registered for the study. There were 33 patients with newly diagnosed adult extranodal NKTCL enrolled from 6 study sites in China during the clinical trial period from 2012 to 2013. Among these enrolled patients, 19 were from Beijing Cancer Hospital, seven from the Fourth Hospital of Hebei Medical University, two from Peking University Third Hospital, two from the 307 Hospital of PLA, two from the Tumor Hospital of Shanxi Province, and one from the Peking University First Hospital. There were 19 males and 14 females with a median age of 39 years (range 19–64 years). Due to the rarity of this disease, it was difficult to adequately randomize patients for this trial. However, this study represents the largest patient cohort enrolled in a trial in Asia.

### PEG-L-CHOP regimen

The PEG-L-CHOP treatment procedure included cyclophosphamide 750 mg/m^2^ intravenously on day 1, vincristine 1.4 mg/m^2^ intravenously on day 1 (the single maximum dose 2 mg), adriamycin 50 mg/m^2^ intravenously on day 1, prednisone 100 mg on days 1–5, and PEG-ASP (3750 units/vial, Jiangsu Hengrui Medicine Co., Ltd., Jiangsu, China) 2500 IU/m^2^ intramuscular injection on day 2 (the single maximum dose 3750 units).

### Patient treatment assessments

The primary endpoint was the overall responsive rate (ORR) and CR. The secondary endpoint was the adverse reaction. We evaluated the available patient information including history, physical examination, complete hematological and biochemical tests, CT scans of neck, chest, abdomen and pelvic, or 18 fluorodeoxyglucose positron emission tomography, bone marrow aspirate and biopsy. The clinical staging was performed according to the Ann Arbor classification system. Efficacy evaluation was conducted after two cycles of PEG-L-CHOP according to the 2007 revised guidelines for response criteria for malignant lymphoma [25]. The lesion responses were classified as complete remission (CR), unconfirmed complete remission (Cru), partial remission (PR), and progressive disease (PD). Adverse events were graded according to the US National Cancer Institute Common Terminology Criteria for Adverse Events (NCI CTCAE), version 4.0. During the study, various adverse events (AE) and serious adverse events (SAE) were recorded. The relationship about between AE and the tested drug was assessed and designed as certain, probably, doubtful, unrelated and not assessable.

### Sample methods and statistical analysis

#### Sample size

This study enrolled 33 eligible patients. The CR rate for standard CHOP regimen plus radiation therapy for previously untreated ENKTL patients has been reported to be 27% following [8]. The CR rate increases to 81.6% with CHOP-L (cyclophosphamide, vincristine, doxorubicin, dexamethasone and *Escherichia coli* L-asparaginase) [26]. Therefore, a target CR rate of 81.6% with PEG-L-CHOP regimen was used to calculate the sample size. With a statistical power of 90% and a one-sided, type I error of 1%, the number of eligible patients required for this study was calculated to be 16. With an estimated dropout rate at 10%, the total sample size needed was calculated to be18 patients per treatment.

#### Statistical analysis

Data was analyzed using SAS 9.2 software. The correlation between clinical, pathological factors and CR was evaluated using Two-tailed chi-squared test (χ^2^) or Fisher’s exact test. Multi-factor logistic regression was performed to adjust for possible confounding factors and to calculate odds ratios. The 1, 2, or 3-year survival rates were analyzed using the Kaplan–Meier method for log-rank comparison of survival between groups. Prognostic factors affecting the survival rate were also assessed using the log-rank test. *p* < 0.05 was considered statistically significant. Variables with *p* < 0.05 in the univariate logistic model were included in the multiple logistic regression model. The *p* values in Table 2 were derived from the two-tailed chi-squared (χ2) or Fisher’s exact comparisons, and the univariate logistic regression. The four variables (Arbor Stage, LDH level, IPI score, and number of extranodal involvement sites) were included in the final model calculation. However, the small sample sizes in several categories in Table 2 lowered the model reliability below the validity level, therefore, the multivariate analysis data are not shown. Univariate analysis showed that ECOG and IPI affected the prognosis (*p* < 0.05), while the other clinical factors did not affect the prognosis. These two factors are all included in the multiple factor survival analysis, and there are not effective independent factors found.

## Results

### Clinical characteristics of patients with Extranodal NK/T cell lymphoma

In this non-randomized clinic trial, we enrolled eligible patients at 6 participating institutions in China between January 2012 and July 2013. The 33 patients with newly diagnosed adult extranodal NK/T lymphoma included 30 (90.9%) upper aero-digestive tract NK/T-cell lymphoma (UNKTCL), and 3 (9.1%) extra-upper aero-digestive tract NK/T-cell lymphoma (EUNKTCL). Twenty-one patients (63.6%) were Ann Arbor stage I~II, and 12 patients (36.4%) patients were stage III ~ IV. International Prognostic Index score (IPI) was 1 or lower in 22 patients (66.7%) and 2 or higher in 11 patients (33.3%). B symptoms were observed in 12 patients (36.4%). In this group of patients, the most common clinical symptoms were nasal obstruction and rhinorrhea (87.9%), and some of the patients had bloody nasal discharge. The detail clinical characteristics of the group are listed in Table 1.

**Table 1:** Clinical Characteristics of Patients with newly Diagnosed Adult NKTCL (*n* = 33)

Characteristic	Patients	
	No	%
Age		
< 60 years	30	90.9
≥ 60 years	3	9.1
Gender		
Male	19	57.6
Female	14	42.4
Primary site		
UNKTCL	30	90.9
EUNKTCL	3	9.1
An Arbor stage		
I~II	21	63.6
III~IV	12	36.4
B symptoms		
Yes	12	36.4
No	21	63.6
ECOG		
0–1	27	81.8
≥ 2	6	18.2
LDH		
Increased	7	21.2
Normal	26	78.8
IPI score		
0~ 1	22	66.7
≥ 2	11	33.3
β2-microglobulin		
Increased	8	24.2
Normal	25	75.8
Bone marrow involvement		
Yes	4	12.1
No	29	87.9
Lymph node involvement		
Yes	12	36.4
No	21	63.6
Extranodal involvement site		
0~ 1	26	78.8
≥ 2	7	21.2

*Abbreviations: UNKTCL* upper aerodigestive tract NK/T-cell lymphoma, *EUNKTCL* non-upper aerodigestive tract NK/T-cell lymphoma, *LDH* lactate dehydrogenase, *ECOG* Eastern Cooperative Oncology Group, *IPI* international prognostic index

### PEG-L-CHOP therapy outcomes

All 33 patients received the PEG-L-CHOP chemotherapy in a total of 170 cycles of treatment. The mean treatment cycle in this study was 5.2 (range 1–6 cycle). Sixteen patients received localized tumor radiotherapy with a radiation dose of 50 Gy. We performed the CR assessment at the completion of chemoradiotherapy. The overall responsive rate (ORR) was 96.9% (32/33). Twenty-five patients (75.8%) achieved complete remission (CR), 7 patients (21.2%) achieved partial remission (PR), and 1 patient (3%) had progressive disease (PD). We further identified that CR rate was significantly higher (90.5%, *p* = 0.015) in patients with stage I-II disease (19 /21 patient) compared with patients with stage III-IV disease who had a CR rate of 50.0% (6/12). In addition, in our multivariable regression model, we found that patients with increased lactate dehydrogenase, IPI score of 2 or higher, and greater extranodal distribution sites to be significantly associated with a lower CR (*p* < 0.05) (Table 2). Clinical factors including age, gender, primary site, B symptoms, ECOG, and β2-microglobulin, however, did not affect the PEG-L-CHOP chemotherapy outcomes (Table 2).

**Table 2:** Clinical Characteristics and Therapeutic Efficacy in Patients with Newly Diagnosed Adult NKTCL (*n* = 33)

Characteristic	Patients (%)	CR (%)	NO CR (%)	*p* value
Age				
<60 years	30 (90.9)	23 (76.7)	7 (23.3)	1.000
≥ 60 years	3 (9.1)	2 (66.7)	1 (33.3)	
Gender				
Male	19 (57.6)	16 (84.2)	3 (15.8)	0.238
Female	14 (42.2)	9 (64.3)	5 (35.7)	
Primary site				
UNKTCL	30 (90.9)	23 (76.7)	7 (23.3)	1.000
EUNKTCL	3 (9.1)	2 (66.7)	1 (33.3)	
An Arbor Stage				
I~II	21 (63.6)	19 (90.5)	2 (9.5)	0.015*
III~IV	12 (36.4)	6 (50.0)	6 (50.0)	
B symptom				
Yes	12 (36.4)	9 (75.0)	3 (25.0)	1.000
No	21 (63.6)	16 (76.2)	5 (23.8)	
ECOG				
0–1	27 (81.8)	21 (77.8)	6 (22.2)	0.616
≥ 2	6 (18.2)	4 (66.7)	2 (33.3)	
LDH				
Increased	7 (21.2)	2 (28.6)	5 (71.4)	0.004*
Normal	26 (78.8)	23 (88.5)	3 (11.5)	
IPI score				
0~ 1	22 (66.7)	21 (95.5)	1 (4.5)	< 0.001*
≥ 2	11 (33.3)	4 (36.4)	7 (63.6)	
β2-microglobulin				
Increased	8 (24.2)	5 (62.5)	3 (37.5)	0.366
Normal	25 (75.8)	20 (80.0)	5 (20.0)	
BM involvement				
Yes	4 (12.1)	3 (75.0)	1 (25.0)	1.000
No	29 (87.9)	22 (75.9)	7 (24.1)	
Lymph node involvement				
Yes	12 (36.4)	9 (75.0)	3 (25.0)	1.000
No	21 (63.6)	16 (76.2)	5 (23.8)	
Extranodal involvement site				
0–1	26 (78.8)	22 (84.6)	4 (15.4)	0.042
≥ 2	7 (21.2)	3 (42.9)	4 (57.1)	
Allergy history				
Yes	1 (3.0)	0 (0.0)	1 (100.0)	0.242
No	32 (97.0)	25 (78.1)	7 (21.9)	

*Abbreviations: CR* complete remission, *No CR included PR* partial remission, *SD* stable disease and *PD* progressive disease

*Fisher’s exact test, sample sizes in some cells are 5 or less

### Post treatment follow up

**Fig. 1:**
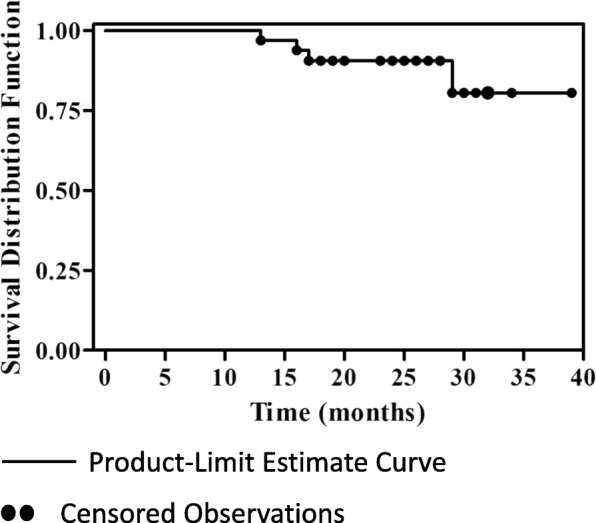
Overall survival curve in 33 patients with adult extranodal NK/T cell lymphoma. The patients were treated with PEG-L-CHOP every 21 days for 6 cycles. Follow-up data was collected and Kaplan-Meier survival analyses were performed. The overall survival rate at 1, 2, and 3 year was 100, 90.61, and 80.54%, respectively

**Fig. 2:**
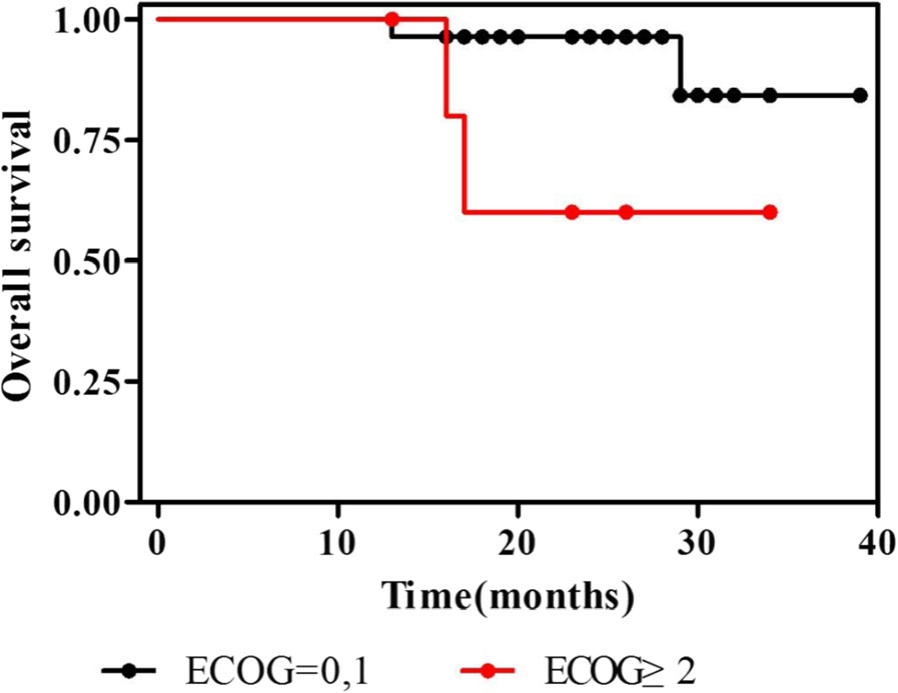
Survival curves for all patients by Eastern Cooperative Oncology Group performance status (ECOG). The patients were treated with PEG-L-CHOP every 21 days for 6 cycles. Survival analyses were performed for ECOG using Kaplan-Meier method. Patients with ECOG performance of 2 or high showed less survival rate that patients with ECOG performance of 0~ 1. *p* = 0.0278 by log-rank test

**Fig. 3:**
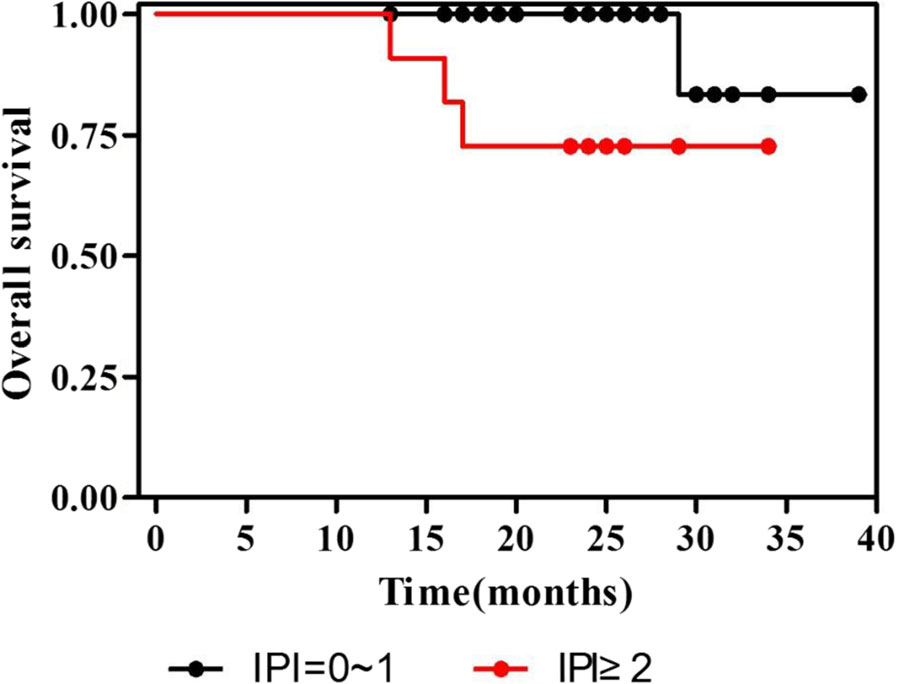
Survival curves for all patients according to international prognostic index (IPI). The patients were treated with PEG-L-CHOP every 21 days for 6 cycles. Survival analyses were performed according to IPI score using Kaplan-Meier method. Patients with IPI score of 2 or higher had poor efficacy than patients with IPI score 0~ 1. *p* = 0.0186 by log-rank test

We were able to collect follow-up data on all 33 patients enrolled in our trial. Four patients died of tumor relapse and progression posttreatment. The first patient, who had primary intestinal NKTCL, reached achieved CR at the end of therapy, but died of tumor relapse 5 months after the end of the clinical trial. The second patient with stage IV lymphoma and extensive systemic invasion suffered disease progression after six cycles treatments. This patient died due to invalid rescue treatment. The other two patients had stage II lymphoma at diagnosis and did not reach CR after treatment. The median overall survival time has not been obtained. The overall survival rate at 1, 2, and the 3 years was 100, 90.61, and 80.54%, respectively for the entire treatment cohort. Statistical analysis showed that only ECOG and IPI affected the prognosis (*p* < 0.05) (Figs. 1, 2 and 3).

### Safety and tolerability of PEG-L-CHOP therapy

We observed adverse side reactions in all patients enrolled in the study (Table 3). There were no PEG-L-CHOP therapy-related deaths or allergic reactions during treatment. Most adverse reactions were grade I-II. Most patients experienced bone marrow suppression (78.8%). Twenty-one patients experienced grade III~IV neutropenia (63.6%). Nine patients (27.2%) had thrombocytopenia (among them, 2 (6%) were grade III~IV). Twenty patients (60.6%) had liver dysfunction. One patient with grade III~IV liver dysfunction normalized their liver function tests after treatment. We also observed grade I-II fibrinogen reduction in 15 patients (45.5%) without any associated clinical occurrence of mucocutaneous or visceral hemorrhage. There were four cases of infections including with a case each of herpes simplex, pneumonia, gingivitis, and diarrhea induced septic shock. The first three cases of infection completed the full treatment, while the patient with septic shock withdrew from the trial and recovered following cessation of therapy. Other common adverse reactions included nausea, vomiting, and anemia (Table 3).

**Table 3:** Adverse Events in the Patients with Adult NKTCL (*n* = 33)

Common adverse event	I	II	III	IV	*n* (%)
Systemic reactions					
Allergic reaction	0	0	0	0	0 (0.0)
Drug fever	0	0	0	0	0 (0.0)
Infection	2	1	1	0	4 (12.1)
Hyperglycemia	3	0	0	0	3 (9.1)
Coagulation function					
Decreased fibrinogen	14	1	0	0	15 (45.5)
Prolonged APTT					^a^19(57.6)
Prolonged PT					^a^9 (27.3)
Thrombosis	0	1	0	0	1 (3.0)
Hemorrhage	0	0	0	0	0 (0.0)
Blood system					
Neutropenia	2	2	7	14	25 (75.8)
Anemia	8	3	1	0	12 (36.4)
Thrombocytopenia	5	2	1	0	8 (24.2)
Gastrointestinal tract					
Increase in ALT/AST	17	2	3	0	22 (66.7)
Increase in bilirubin	11	0	0	0	11 (33.3)
Pancreatitis	0	0	0	0	0 (0.0)
Vomiting	4	4	0	0	8 (24.2)
Diarrhea	0	0	1	0	1 (3.0)
Respiratory system					
Cough	0	0	0	0	0 (0.0)
Chest tightness	0	0	0	0	0 (0.0)
Bronchial spasm	0	0	0	0	0 (0.0)
Cardiovascular system					
Arrhythmia	0	0	0	0	0 (0.0)
Hypotension	0	0	0	0	0 (0.0)
Cardiac insufficiency	0	0	0	0	0 (0.0)
Nervous system					
Headache	0	0	0	0	0 (0.0)
Insomnia	0	0	0	0	0 (0.0)
Mental change	0	0	0	0	0 (0.0)
Urinary system					
Renal damage	0	0	0	0	0 (0.0)
Urine color change	0	0	0	0	0 (0.0)
Other events	1	1	0	0	2 (6.0)

Other events included hair loss in 2 cases. ^a^Adverse events not graded in the NCI CTCAE classification

## Discussion

The clinical application of PEG-ASP to treat adult and pediatric acute lymphoblastic leukemia (ALL) patients began half a century ago. However, optimal treatment outcomes between adult and pediatric cancers are not well characterized. Two major limitations of L-ASP are the development of hypersensitivity (15 to 73%) and the need for frequent intravenous dosing [27]. PEG-ASP decreases the immunogenicity and the risk of hypersensitivity reactions [27]. The comparative study between native L-asparaginase (L-ASP) and PEG-ASP treatments in children ALL patients suggested that PEG-ASP treatment had a more rapid clearance of tumor cells and more durable asparaginase activity compared with L-asparaginase (L-ASP) [27]. However, the half-life of L-ASP is only 26 h [27]. The serum asparaginase level (≥ 0.1 IU/mL) that existed in 95% patients at day 11 of therapy was quickly reduced to 88% by day 18, and to 7% by day 25 after a single dose of intravenous PEG-ASP (2500 IU/m^2^) [27] . When comparing L-ASP and PEG-ASP, both drugs have similar patient survival rates, adverse events, and infection occurrence rates [27], therefore, PEG-ASP has become the first line treatment for ALL [14]. PEG-ASP effectively depleted the asparagine in the blood of adult ALL patients with an associated increase in the CR rate to 77% [14].

L-ASP has been an important drug for the treatment of adult NKTCL. Standard therapy with CHOP plus radiation for untreated ENKTL only achieved CR rates in previously reported studies [8]. It has been reported that adults patients with NKTCL treated with an L-ASP-containing regimen of dexamethasone, methotrexate, ifosfamide, L-ASP, and etoposide (SMILE) achieved an 81% overall response rate (ORR) (CR =66%, PR =15%) and 50% 5-year total survival rate [28]. However, this treatment caused strong negative side effects and was associated with mortality in 6.9% of patients (6/87, 5 sepsis-related deaths and 1 acute renal failure associated death) [28]. Several other studies have used PEG-ASP in combination with P-GEMOX(PEG-ASP, Gemcitabine,and Oxaliplatin)regimen to treat advanced stages or relapsed/ refractory NKTCL. In these studies, the ORR ranged between 73.7–80.0%, and the 3-year overall survival rate (OS) was 64.7% [29]. The ORR further increased to about 92.1–96.3%, and 2-year OS was 86.0% with the combination of gemcitabine, oxaliplatin, and L-asparaginase (GELOX) followed by radiation therapy for patients with stage IE/IIE ENKTL [30, 31]. Several studies have demonstrated that cyclophosphamide increases NK cell activity and plays a role in anti-tumor in vitro and in vivo [3, 32]. There is no standard chemotherapy regimen for patients with ENTCL. We have begun to adopt L-ASP and cyclophosphamid containing regimens(CHOP)to treat 45 patients with relapsed or refractory NK/T cell lymphoma from 1996 to 2008. We obtained 82.2% ORR (CR = 55.6%) and 66.7% 5-year total survival rate [8]. Our findings suggested that L-ASP combination CHOP (L-CHOP) was an effective treatment for the patients with refractory and relapsed extranodal NKTCL [8]. We have further applied L-ASP in combination with CHOP to treat 38 newly-diagnosed NKTCL from 2008 to 2012 [26]. We therefore carried out the prospective study to evaluate the efficacy and safety of PEG-ASP in combination with CHOP and radiation in the treatment of newly-diagnosed NKTCL in Chinese patients. In this study, we used PEG-ASP treatment at a dose of 2500 IU/m^2^ intramuscular injection on day 2 (the single maximum dose 3750 units) based on the drug specific prescription guideline, our previous findings [23], and other reports [14, 20, 24, 27]. The mean BSA was 1.69 (range 1.3–2). In the present study, PEG-asparaginase activity levels were not monitored at the time of study due to the lack of instrumentation.

In a recent trial, Youssef et al. enrolled 23 patients with adult untreated ENKTCL which included young patients (median age of 38 years old) patients with upper aero-digestive tract disease, with symptoms of nasal obstruction, rhinorrhea, and nasal discharge with blood. They concluded that nasal type NK/T-cell lymphoma with poor response to the conventional anthracycline-based chemotherapy needed a novel therapy to improve survival [33]. Our findings had the similar conclusions to these reports [33, 34]. In our current study, patients NKTCL distributions were very different to the above reports. For example, the diagnosed patients with NKTCL were outside the nasal cavity, mainly involving the skin and gastrointestinal tract, following the patients with lesions in bone marrow, liver, and spleen. About 1/3 patients were at stage III ~ VI, with B symptoms, and IPI score was a moderate-high risk or high risk. We treated patients with PEG-L-CHOP and achieved 96.9% ORR (75.8% CR and 21.2% PR). This treatment efficacy was similar to our previous report when we used L-ASP combined with CHOP regimen for treatment of NK/T cell lymphoma [26] and was much higher efficacy than other reports that combined chemotherapy regimen based on doxorubicin [6, 35, 36]. Moreover, our overall group survival rates were higher which were 100, 90.61, and 80.54%, at 1, 2, and 3 years respectively. There were four patients who died of disease progression from tumor relapse in our follow-up study. In the future treatments, we speculate that the application of several new drugs which target immune checkpoints such as PD-1, PDL-1 and CTLA4 may enhance the host anti-tumor immune response as reported [37]. In this clinical trial, we observed lower survival rates in patients with high IPI and high ECOG. We speculate that patients with high ECOG do not tolerate PEG-L-CHOP well, and patients with high IPI require more aggressive regimens (e.g. SMILE), or new drugs. Epstein-Barr virus (EBV) was the first identified human oncogenic DNA virus in the gamma-herpesvirus family. Several reports suggested the association between Epstein Bar Viruses (EBV) and NK/T cell lymphoma [38–41]. It has been suggested that the EBV-DNA level in plasma was a good indicator for response and overall survival in nasal type NK/T-cell lymphoma. Three-year overall survival rate for plasma EBV-DNA positive and negative patient was 42.9 and 94.4%, respectively [41]. However, we did not measure EBV-DNA level at the time of this study, but we have more recently added routine serum and tumor EBV-DNA detection in pre- and post- treatment patients. Future studies evaluating effects of PEG-L-CHOP in EBV associated ENKTCL is warranted.

In our study, there were no treatment-related deaths. We found that the adverse reactions mostly graded I~II in the 33 patients with adult NKTCL treated with PEG-L-CHOP. The most common adverse reactions were bone marrow suppression, followed by elevated transaminases and bilirubin. These adverse events were similar to those observed in the L-ASP combined with CHOP regimen in our previous report [26]. Previous studies found that PEG did not increase the rate of myelosuppression/ gastrointestinal toxicities [21]. However, PEG-asparaginase (PEG-ASP)treatment caused hepatotoxicity in ALL patients [42]. CHOP treatment was independently associated with myelosuppression and gastrointestinal toxicities, and PEG-ASP treatment caused additional hepatotoxicity, hypofibrinogenemia, hyperglycemia, hypertriglyceridemia, pancreatitis, thrombosis, and hemorrhage [13, 18, 26, 27, 31]. In this study, there were 3 (grade I) hyperglycemia episodes in our 33-patient cohort. However, there were no symptoms of hyperlipidemia or pancreatitis. We speculated that these observations related to the low-fat diets during the patient treatments. We found the increasing level of liver function ALT/AST (grade 1–2, tolerable) in 66.7% patients, and increasing bilirubin levels (grade I) in 33.3% (11 out of 33) of treated patients. These symptoms were relieved 1–2 weeks post treatment cessation. Patients who experienced abnormalities in their liver function tests (LFTs) were older and had a high body surface area (BSA), but they were able to complete therapy despite these low grade abnormalities in their LFTs. Based on our clinical and research experience and observations, we found that there was no increased rate of myelosuppression/ gastrointestinal toxicity in PEG-ASP and CHOP combination therapy compared with standard CHOP for NKTCL treatment.

The incidence of allergic reactions to L-ASP treatment regimen has been reported to be approximately 15 to 73% [15, 16]. Hypersensitivity is derived from the L-ASP antibody reactions that reduce plasma asparaginase activity, leading to the development of drug resistance [27]. The patient serum antibody titer to PEG-ASP was only about 2%, much lower than 26% titer to L-ASP in the first intensification phase [27]. High-titer antibodies were associated with low asparaginase activity in the native L-ASP structure [27]. Hypersensitivity often develops without clinical manifestation, or so called “silent” hypersensitivity. “Silent” hypersensitivity has been reported in NKTCL treated patients that received bacterial derived PEG-ASP [43]. Therefore, any non-clinical manifestations of allergic reactions to PEG-ASP must be considered. For example, there was an approximately 4.4% allergic reaction rate for PEG-ASP regimen treatment in a Chinese clinical study of 135 children with ALL [44]. We have not observed clinical allergic reactions among our 33 patients for all 170 treatments of PEG-ASP. Each patient has received a dose of dexamethasone (10 mg IV) before PEG-ASP. We speculate that this procedure reduces the clinical hypersensitivity. In addition, the administration of PEG-asparaginase by intravenous (IV) or intramuscular (IM) IV may affect clinical allergic reactions. For example, it has been reported that administering PEG-ASP through IM had significantly lower risk of hypersensitivity compared to IV [44]. In future studies, we will monitor drug concentrations and antibody development to further optimize the treatment. Further studies to matched comparison between historic control receiving CHOP and a propensity score may provide the appropriately validation to conclude that PEG-CHOP improves outcomes compared to CHOP alone.

## Conclusion

In summary, based on the current clinical trial, we demonstrate that PEG-ASP combined with CHOP (PEG-L-CHOP) was both safe and effective in the treatment of adult patients with untreated ENKTCL. The major advantages of the PEG-L-CHOP regimen compared to the L-CHOP regimen include fewer allergic reactions, convenient dosing schedule (single dose of PEG-ASP versus seven consecutive doses of L-ASP administered daily), and shortened hospitalization stays. Further studies with a larger patient cohort, patient randomization and double-blinded study design may provide more meaningful conclusions to assess the benefits of PEG-ASP-based regimens in the treatment of NKTCL.

## Abbreviations

ALL: acute lymphoblastic leukemia
ASNS: asparagines synthetase
CHOP: Cyclophosphamide, hydroxydaunorubicin (doxorubicin), oncovin (vincristine), and prednisolone
CHOP-L: L-ASP- based regimen
CY: Cyclophosphamide
EBV: Epstein Bar Viruses
IM: intramuscular
IPI: international prognostic index
IV: intravenously
L-ASP: L-asparaginase
NCCN: National Comprehensive Cancer Network
NKTCL: Extranodal natural killer (NK)/T-cell lymphoma
ORR: overall response rate
OS: overall survival
PEG-ASP: polyethylene glycol-conjugated asparaginase
PEG-L-CHOP: PEG-ASP combined with CHOP
SMILE: regimen of dexamethasone, methotrexate, ifosfamide, L-asparaginase, and etoposide
UNKTCL: upper aerodigestive tract NK/T-cell lymphoma

## References

[1] Kohrt H, Advani R (2009). Extranodal natural killer/T-cell lymphoma: current concepts in biology and treatment. Leuk Lymph.

[2] Kwong YL (2005). Natural killer-cell malignancies: diagnosis and treatment. Leukemia.

[3] Sharma B, Vaziri ND (1984). Augmentation of human natural killer cell activity by cyclophosphamide in vitro. Cancer Res.

[4] Tse E, Kwong YL (2013). How I treat NK/T-cell lymphomas. Blood.

[5] Lee J, Suh C, Park YH, Ko YH, Bang SM, Lee JH, Lee DH, Huh J, Oh SY, Kwon HC (2006). Extranodal natural killer T-cell lymphoma, nasal-type: a prognostic model from a retrospective multicenter study. J Clin Oncol.

[6] Li CC, Tien HF, Tang JL, Yao M, Chen YC, Su IJ, Hsu SM, Hong RL (2004). Treatment outcome and pattern of failure in 77 patients with sinonasal natural killer/T-cell or T-cell lymphoma. Cancer.

[7] Yamaguchi M (2012). Current and future management of NK/T-cell lymphoma based on clinical trials. Int J Hematol.

[8] Yong W, Zheng W, Zhu J, Zhang Y, Wang X, Xie Y, Lin N, Xu B, Lu A, Li J (2009). L-asparaginase in the treatment of refractory and relapsed extranodal NK/T-cell lymphoma, nasal type. Ann Hematol.

[9] Suzuki R (2012). NK/T-cell lymphomas: pathobiology, prognosis and treatment paradigm. Curr Oncol Rep.

[10] Kim M, Kim TM, Kim KH, Keam B, Lee SH, Kim DW, Lee JS, Jeon YK, Kim CW, Heo DS (2015). Ifosfamide, methotrexate, etoposide, and prednisolone (IMEP) plus L-asparaginase as a first-line therapy improves outcomes in stage III/IV NK/T cell-lymphoma, nasal type (NTCL). Ann Hematol.

[11] Suzuki R (2014). Pathogenesis and treatment of extranodal natural killer/T-cell lymphoma. Semin Hematol.

[12] Avramis VI, Panosyan EH (2005). Pharmacokinetic/pharmacodynamic relationships of asparaginase formulations: the past, the present and recommendations for the future. Clin Pharmacokinet.

[13] Earl M (2009). Incidence and management of asparaginase-associated adverse events in patients with acute lymphoblastic leukemia. Clin Adv Hematol Oncol.

[14] Wetzler M, Sanford BL, Kurtzberg J, DeOliveira D, Frankel SR, Powell BL, Kolitz JE, Bloomfield CD, Larson RA (2007). Effective asparagine depletion with pegylated asparaginase results in improved outcomes in adult acute lymphoblastic leukemia: Cancer and leukemia group B study 9511. Blood.

[15] Zeidan A, Wang ES, Wetzler M (2009). Pegasparaginase: where do we stand?. Expert Opin Biol Ther.

[16] Silverman LB, Supko JG, Stevenson KE, Woodward C, Vrooman LM, Neuberg DS, Asselin BL, Athale UH, Clavell L, Cole PD (2010). Intravenous PEG-asparaginase during remission induction in children and adolescents with newly diagnosed acute lymphoblastic leukemia. Blood.

[17] Dinndorf PA, Gootenberg J, Cohen MH, Keegan P, Pazdur R (2007). FDA drug approval summary: pegaspargase (oncaspar) for the first-line treatment of children with acute lymphoblastic leukemia (ALL). Oncologist.

[18] Douer D, Yampolsky H, Cohen LJ, Watkins K, Levine AM, Periclou AP, Avramis VI (2007). Pharmacodynamics and safety of intravenous pegaspargase during remission induction in adults aged 55 years or younger with newly diagnosed acute lymphoblastic leukemia. Blood.

[19] Stock W, Douer D, DeAngelo DJ, Arellano M, Advani A, Damon L, Kovacsovics T, Litzow M, Rytting M, Borthakur G (2011). Prevention and management of asparaginase/pegasparaginase-associated toxicities in adults and older adolescents: recommendations of an expert panel. Leuk Lymph.

[20] Kim HJ, Ock CY, Kim TM, Lee SH, Lee JY, Jung SH, Cho YS, Kim M, Keam B, Kim DW, et al*.* Comparison of native Escherichia Coli L-Asparaginase versus Pegylated Asparaginase, in combination with Ifosfamide, methotrexate, etoposide, and prednisolone (IMEP), in Extranodal NK/T cell lymphoma, nasal type (NTCL). Cancer Res Treat. 2018;50(3):670–80. 10.4143/crt.2017.051 PMC6056977 28675023

[21] Li L, Zhang C, Zhang L, Li X, Wu JJ, Sun ZC, Fu XR, Wang XH, Chang Y, Wang R (2014). Efficacy of a pegaspargase-based regimen in the treatment of newly-diagnosed extranodal natural killer/T-cell lymphoma. Neoplasma.

[22] Li Y, Zhang X, Hu T, Han L, Li R, Wen J, Zhang M (2014). Asparagine synthetase expression and its potential prognostic value in patients with NK/T cell lymphoma. Oncol Rep.

[23] Ping LY, Zheng W, Wang XP, Xie Y, Lin NJ, Tu MF, Ying ZT, Zhang C, Liu WP, Deng LJ (2012). Safety and adverse event profiling of pegylated L-asparaginase combined chemotherapy in the treatment of lymphoma. Zhonghua Yi Xue Za Zhi.

[24] Wen JY, Li M, Li X, Chen J, Lin Q, Ma XK, Dong M, Wei L, Chen ZH, Wu XY (2014). Efficacy and tolerance of pegaspargase-based chemotherapy in patients with nasal-type extranodal NK/T-cell lymphoma: a pilot study. Asian Pac J Cancer Prev.

[25] Cheson BD, Pfistner B, Juweid ME, Gascoyne RD, Specht L, Horning SJ, Coiffier B, Fisher RI, Hagenbeek A, Zucca E (2007). Revised response criteria for malignant lymphoma. J Clin Oncol.

[26] Lin N, Song Y, Zheng W, Tu M, Xie Y, Wang X, Ping L, Ying Z, Zhang C, Deng L (2013). A prospective phase II study of L-asparaginase- CHOP plus radiation in newly diagnosed extranodal NK/T-cell lymphoma, nasal type. J Hematol Oncol.

[27] Avramis VI, Sencer S, Periclou AP, Sather H, Bostrom BC, Cohen LJ, Ettinger AG, Ettinger LJ, Franklin J, Gaynon PS (2002). A randomized comparison of native Escherichia coli asparaginase and polyethylene glycol conjugated asparaginase for treatment of children with newly diagnosed standard-risk acute lymphoblastic leukemia: a Children's Cancer group study. Blood.

[28] Kwong YL, Kim WS, Lim ST, Kim SJ, Tang T, Tse E, Leung AY, Chim CS (2012). SMILE for natural killer/T-cell lymphoma: analysis of safety and efficacy from the Asia lymphoma study group. Blood.

[29] Wang JH, Wang L, Liu CC, Xia ZJ, Huang HQ, Lin TY, Jiang WQ, Lu Y (2016). Efficacy of combined gemcitabine, oxaliplatin and pegaspargase (P-gemox regimen) in patients with newly diagnosed advanced-stage or relapsed/refractory extranodal NK/T-cell lymphoma. Oncotarget.

[30] Jing XM, Zhang ZH, Wu P, Zhang SC, Ren YR, Xiong ZJ, Wei W, Luo L, Li L (2016). Efficacy and tolerance of pegaspargase, gemcitabine and oxaliplatin with sandwiched radiotherapy in the treatment of newly-diagnosed extranodal nature killer (NK)/T cell lymphoma. Leuk Res.

[31] Wang L, Wang ZH, Chen XQ, Li YJ, Wang KF, Xia YF, Xia ZJ (2013). First-line combination of gemcitabine, oxaliplatin, and L-asparaginase (GELOX) followed by involved-field radiation therapy for patients with stage IE/IIE extranodal natural killer/T-cell lymphoma. Cancer.

[32] Stewart LS, Sewell HF, Thomson AW (1990). Combination chemo-immunotherapy: kinetics of in vivo and in vitro generation of natural killer cells and lymphokine-activated killer cells in the rat. Clin Exp Immunol.

[33] Youssef YB, Bougmiza I, Bouabid Z, Achour B, Regaieg H, Sriha B, Belkadhi M, Omri HE, Khelif A (2012). Nasopharyngeal/nasal type NK/T lymphoma: analysis of 23 cases and current review of the literature. Kulak Burun Bogaz Ihtisas Derg.

[34] Miyake MM, Oliveira MV, Miyake MM, Garcia JO, Granato L (2014). Clinical and otorhinolaryngological aspects of extranodal NK/T cell lymphoma, nasal type. BrazJ Otorhinolaryngol.

[35] Yong W, Zheng W, Zhang Y (2001). Clinical characteristics and treatment of midline nasal and nasal type NK/T cell lymphoma. Zhonghua Yi Xue Za Zhi.

[36] Wang B, Lu JJ, Ma X, Guo Y, Lu H, Hong X, Li J (2007). Combined chemotherapy and external beam radiation for stage IE and IIE natural killer T-cell lymphoma of nasal cavity. Leuk Lymphoma.

[37] Mahoney KM, Rennert PD, Freeman GJ (2015). Combination cancer immunotherapy and new immunomodulatory targets. Nat Rev Drug Discov.

[38] Borza CM, Morgan AJ, Turk SM, Hutt-Fletcher LM (2004). Use of gHgL for attachment of Epstein-Barr virus to epithelial cells compromises infection. J Virol.

[39] Klein F, Rosensteel JF, Hummer RM, Hillman EA, Riggs CW, Charmella LJ (1978). Large-scale production and concentration of infectious Epstein-Barr virus. Appl Environ Microbiol.

[40] Shibley GP, Manousos M, Munch K, Zelljadt I, Fisher L, Mayyasi S, Harewood K, Stevens R, Jensen KE (1980). New method for large-scale growth; and concentration of the Epstein-Barr viruses. Appl Environ Microbiol.

[41] Suzuki R, Yamaguchi M, Izutsu K, Yamamoto G, Takada K, Harabuchi Y, Isobe Y, Gomyo H, Koike T, Okamoto M (2011). Prospective measurement of Epstein-Barr virus-DNA in plasma and peripheral blood mononuclear cells of extranodal NK/T-cell lymphoma, nasal type. Blood.

[42] Ogawa C, Manabe A, Ohara A, Ishiguro A (2013). Current national and international status of supportive therapy for the coagulopathy associated with L-asparaginase containing regimen for acute lymphoblastic leukemia. Rinsho ketsueki.

[43] Neumann DR, Marini BL, Phillips TJ, Wilcox RA, Mayer TL, Brown A, Perissinotti AJ. Pegasparaginase silent inactivation during therapy for NK/T cell lymphoma. Leuk Lymphoma. 2018;59(7):1596–605. 10.1080/10428194.2017.1393672 29105525

[44] Cooperation Group of Phase IICToPEGA (2008). Comparison of polyethylene glycol conjugated asparaginase and L-asparaginase for treatment of childhood acute lymphoblastic leukemia. Zhonghua Xue Ye Xue Za Zhi.

